# A Reply to “Was Hahnemann Inspired?”

**Published:** 1888-05

**Authors:** D. C. McLaren

**Affiliations:** Nashville, Mich.


### A REPLY TO "WAS HAHNEMANN INSPIRED?"

Dear Editors: Under the caption "Was Hahnemann Inspired?" Dr. Van Denburg makes some very surprising statements. As these have passed unremarked in either the February or March numbers, I feel it my duty as a student of the *Organon* and a faithful practitioner of the Homœopathy of Hahnemann to offer a reply, however feeble or faltering.

To the remarks on mesmerism a few sentences from Section 293 ought to be a sufficient rejoinder. Hahnemann says this curative power differs greatly from all other remedies, and of its "efficacy none but madmen can entertain a doubt," and further, that it acts homœopathically by exciting symptoms analagous to those of the malady. It acts likewise by imparting a uniform degree of vital power to the organism when there is an excess of it at one point and a deficiency at another; finally, it acts by immediately communicating a degree of vital power to a weak part or to the entire organism—an effect that cannot be produced by any other means with such certainty, and without interfering with the other medical treatment. In note 2 he adds—"this positive communication of the vital power is no more a palliative than food or drink to hunger and thirst."

The Doctor's talk about Morphia in the treatment of renal calculus betrays a great lack of experience and a complacent indifference toward the comparative results of homœopathic and allopathic practice. Who has not known of many such cases suffering agonies day after day in spite of all the Opium and Morphia the allopaths dare administer? And yet it is asserted, without any reservation whatever, and with the air almost of omniscience, "that the proper amount of Morphia will inside of ten minutes begin to ease the brain." Possibly the last word was a misprint for pain; but the whole article looks like an endeavor on the part of the Doctor to ease his brain (or conscience) of sundry trangressions against the law of similars. A few cases will show that the "old veterans" are not all dead yet, or rather that their method still lives and bears the same fruits of success in these latter days.

CASE I.—A homœopathic physician of the uncertain kind fell a victim to gall-stones and suffered untold agonies generally for a week or longer at each attack, the attacks recurring with distressing frequency for two years or more. Unskilled (because untaught) in the correct homœopathic treatment of this malady, and not having any other homœopath in reach, he fell into the hands of the allopaths with the result above stated; finally he wrote asking me the treatment I would recommend. Accordingly I prepared a list of remedies with the indications for each; he had not much difficulty in the choice of a remedy, and when found it cured the case completely, so that for seven years he has been free from trouble of this kind, and for aught I know has enjoyed good health in every other respect. Remedy was China.

CASE II.—An old lady was taken last Thanksgiving Day, after the usual hearty dinner of that anniversary, with severe gall-stone colic. The nearest allopath was summoned, and for the space of seven weeks the stereotyped Morphine treatment was followed; finally, when the patient was apparently at death's door, I was summoned to the case. The skin and eyes were heavily jaundiced, the stomach was so disturbed that only sips of ice-water could be retained; the bowels moved only under the influence of purgatives, and these invariably caused most intense exacerbations of the colic with the poor result of scanty, clay-colored stools; urine full of bile, and the liver extending fully two inches below the ribs, while the gall bladder, enlarged to the size of a man's fist, hung down as far as the iliac crest, feeling quite solid to the touch, and apparently full of thickened bile, if not impacted calculi. A dose of Nux v.$^{200}$ changed the whole aspect of the case in a few hours so that the appetite returned, the vomiting ceased, and patient had a natural stool every day; during the following week a dozen or more gall-stones, averaging a quarter inch in diameter, were passed without pain, and only one attack of colic supervened, about the tenth day after the initial dose; this was promptly met by a dose of Lycopodium$^{10m}$, and convalescence was at once established. The gall bladder emptied itself of its contents and withdrew to its normal position in a few days, while the liver followed more slowly.

CASE III.—Summoned shortly after midnight to a lady in great pain, I found the case to be one of renal colic. She was writhing, moaning, and wringing her hands; the pain culminated every little while in distressing retching and vomiting; there was also constant urging to stool and to urinate with the pain. A dose of Nux v. every five minutes put her to sleep in half an hour.

These cases are emergencies in which a cool head is required to observe symptoms accurately and choose the remedy wisely. Such work can never be done by ignorant physicians. It is sad to think that any such exist to prey upon the public. How long before ignorance in physicians will be legally, as it is morally, criminal?

A word about the "bastinado" case in the same number. Why was not the homœopathic remedy for the effect of anæsthetics used, viz.: Acetic acid or vinegar? Or if used, why not record its failure? D. C. MCLAREN.

NASHVILLE, MICH.

